# Exploring Residents’ Perceptions of Neighborhood Development and Revitalization for Active Living Opportunities

**DOI:** 10.5888/pcd19.220033

**Published:** 2022-09-01

**Authors:** Nishita Dsouza, Natalicio Serrano, Kathleen B. Watson, Jean McMahon, Heather M. Devlin, Stephenie C. Lemon, Amy A. Eyler, Jeanette Gustat, Jana A. Hirsch

**Affiliations:** 1 Department of Community Health and Prevention, Dornsife School of Public Health, Drexel University, Philadelphia, Pennsylvania; 2 Institute for Health Research and Policy, School of Public Health, University of Illinois at Chicago, Chicago, Illinois; 3 Division of Nutrition, Physical Activity, and Obesity, National Center for Chronic Disease Prevention and Health Promotion, Centers for Disease Control and Prevention, Atlanta, Georgia; 4 Division of Emergency Operations, Center for Preparedness and Response, Centers for Disease Control and Prevention, Atlanta, Georgia; 5 UMass Worcester Prevention Research Center, Division of Preventive and Behavioral Medicine, Department of Population and Quantitative Health Sciences, University of Massachusetts Medical School, Worcester, Massachusetts; 6 Department of Epidemiology, Tulane University School of Public Health and Tropical Medicine, New Orleans, Louisiana; 7 Urban Health Collaborative and Department of Epidemiology and Biostatistics, Dornsife School of Public Health, Drexel University, Philadelphia, Pennsylvania

## Abstract

**Introduction:**

Community fears of gentrification have created concerns about building active living infrastructure in neighborhoods with low-income populations. However, little empirical research exists related to these concerns. This work describes characteristics of residents who reported 1) concerns about increased cost of living caused by neighborhood development and 2) support for infrastructural improvements even if the changes lead to a higher cost of living.

**Methods:**

Data on concerns about or support for transportation-related and land use–related improvements and sociodemographic characteristics were obtained from the 2018 SummerStyles survey, an online panel survey conducted on a nationwide sample of US adults (n = 3,782). Descriptive statistics characterized the sample, and χ^2^ tests examined associations among variables.

**Results:**

Overall, 19.1% of study respondents agreed that development had caused concerns about higher cost of living. Approximately half (50.7%) supported neighborhood changes for active living opportunities even if they lead to higher costs of living. Prevalences of both concern and support were higher among respondents who were younger and who had higher levels of education than their counterparts. Support did not differ between racial or ethnic groups, but concern was reported more often by Hispanic/Latino (28.9%) and other non-Hispanic (including multiracial) respondents (25.5%) than by non-Hispanic White respondents (15.6%). Respondents who reported concerns were more likely to express support (65.3%) than respondents who did not report concerns (47.3%).

**Conclusion:**

The study showed that that low-income, racial, or ethnic minority populations support environmental changes to improve active living despite cost of living concerns associated with community revitalization.

**Summary**

**What is already known about this topic?**

New or improved infrastructure in neighborhoods aiming to increase physical activity might lead to unintended social, development, or economic pressures; however, little empirical research exists about residents’ opinions and perceptions of these tradeoffs.

**What is added by this report?**

Our report sheds light on the complexities of public opinions on neighborhood improvements and highlights the need for community engagement on new built environment projects.

**What are the implications for public health practice?**

Participatory planning holds potential for community engagement in decisions about active living infrastructure. Public health and planning professionals can also partner with communities to select the most appropriate measures for each context and evaluate each project for equity and effectiveness.

## Introduction

Regular physical activity positively influences 7 of the 10 most common chronic conditions diagnosed in the US (1). Physical activity improves cognition, decreases depression, and is associated with a reduction in early death and risk for chronic diseases such as coronary heart disease, stroke, type 2 diabetes, obesity, depression, and many forms of cancer (1). Despite the benefits to health, less than half (46%) of US adults engage in enough aerobic physical activity to achieve substantial health benefits (2).

Making physical activity a part of everyday living makes it easier to achieve the benefits of regular physical activity (1). Changes in community design can create opportunities for physical activity and make neighborhoods more supportive of active living (3). The Community Preventive Services Task Force recommends built environment approaches that combine improvements in transportation such as sidewalks, bicycle lanes, and expanded public transit, with changes in land use and community design such as improved parks and recreation facilities and mixed-use development that enable housing in proximity to destinations such as busineses and schools (3).

Communities with low-income populations often have minimal resources for physical activity (4). Furthermore, racial and ethnic minorities, who disproportionately reside in communities with low-income populations, tend to have high rates of leisure-based physical inactivity and chronic diseases such as cardiovascular disease (5). Even when facilities such as parks exist in these communities, they tend to have few amenities, often show neglect, and project a violent or unsafe environment to community residents (6). Geographic health disparities by race, ethnicity, and income have created interest in addressing health equity by improving neighborhood environments through community development (7).

Community development and revitalization strategies that improve neighborhood environments can promote physical activity and improve health in disinvested communities (8). These broad initiatives benefit from multidisciplinary collaboration among health, planning, housing, transportation, and government for the ultimate goal of improving the lives of the community residents (9).

Widespread concerns about gentrification associated with robust community development and revitalization exist (10). However, mixed findings show how active living infrastructure may contribute to these concerns. For example, a study in 3 cities described concerns in communities with Black poulations about the installation of bike lanes and their possible contribution to gentrification (11). Empirical research has found that property values rise as proximity to bicycle facilities increases (12). However, a recent longitudinal analysis of bike lanes showed that these investments were not associated with changing demographics in a neighborhood, and that bike lanes were more commonly installed in neighborhoods with low-income White populations than in neighborhoods with low-income Black populations (13). Contrasting this research are findings that showed that intensive community development such as greenways near downtown areas may increase gentrification (14). Higher property values may encourage gentrification by encouraging long-time homeowners to sell their homes to capture their increased wealth; higher property values can also lead to higher rents, potentially displacing current renters who may no longer be able to afford living in that neighborhood (15). Through this displacement, the remaining residents may lose a sense of belonging in their own neighborhood as their surrounding demographics change, which can negatively affect health and quality of life (10).

Concerns about neighborhood change and potential displacement may present barriers to community support for changes in the built environment to improve access to physical activity. However, limited empirical research exists about residents’ opinions of these potential tradeoffs. Thus, there is a need to understand residents’ perceptions of neighborhood development and revitalization, and their concerns about the potential for displacement of current residents. Our study aimed to describe perceptions and characteristics of residents who reported 1) concerns about increased cost of living from neighborhood development and revitalization and 2) support for neighborhood changes to make it easier to walk or bike even if the changes could lead to a higher cost of living. Because increased cost of living disproportionately affects low-income residents and because racial and ethnic minority populations are overrepresented in disinvested neighborhoods, we hypothesized that low-income and racial and ethnic minority populations would be more likely than non-Hispanic White populations to report concerns about the increased cost of living from neighborhood development and revitalization and less likely to express support for neighborhood changes to make it easier to walk or bike even if the changes could lead to higher cost of living (16).

## Methods

### SummerStyles sample

The 2018 Porter Novelli ConsumerStyles’ database is built from a series of web-based surveys via the GfK KnowledgePanel that gathers insights about US consumers, including information about their lifestyle, health, knowledge, and behaviors (17). Panel members are randomly recruited by using probability-based sampling by address. The panel is continuously replenished and maintains approximately 55,000 panelists. The initial SpringStyles survey was sent from March 21, 2018, to April 11, 2018, to 10,904 panelists. SpringStyles respondents included 6,427 adults who completed the survey for a response rate of 58.9%. Those who completed the SpringStyles survey received reward points worth approximately $5.

Our study used data from the subsequent SummerStyles survey, which was sent to 5,584 respondents that completed SpringStyles, from June 12, 2018, to July 7, 2018. The subsequent SummerStyles survey included survey questions that were not in the initial SpringStyles survey. The final sample had 4,088 adults (response rate = 73.2%). Those who completed the SummerStyles survey also received reward points worth approximately $5. The data were then weighted to match the 2018 US Current Population Survey proportions for sex, age, annual household income, race and ethnicity, household size, education level, census region, and metropolitian statistical area status (18).

Of the 4,088 respondents, we excluded data from 306 respondents (7.4%) who were missing information on concerns and support for active living development (n = 40), physical activity (n = 71), body mass index (n = 64), smoking (n = 98), air pollution (n = 12), and neighborhood features of concern (n = 21). The final analytic sample had data from 3,782 respondents.

### Concerns for neighborhood revitalization and support for walking and biking infrastructure

Respondents were asked about their agreement (using a 5-point Likert scale) with 2 statements: 1) “My neighborhood is experiencing development or revitalization that has caused concerns about higher cost of living”; 2) “I would support changes to my neighborhood to make it easier to walk or bike even if the changes lead to a higher cost of living for me.” Survey questions were developed de novo and were not cognitively tested.

To compare those who agreed there were concerns with those who did not, concern was dichotomized into “concerned” (by grouping “somewhat agree” and “strongly agree”) and “not concerned” (by grouping “strongly disagree,” “somewhat disagree,” and “neither agree nor disagree”). To better distinguish between those who did not support and those who were neutral because the latter group may need different strategies for change, support was categorized into 3 groups: “supporters” (by grouping “somewhat agree” and “strongly agree”), “nonsupporters” (by grouping “strongly disagree” and “somewhat disagree”), and neither (“neither agree nor disagree”). To assess specific concerns, we asked respondents “Which of the following changes to your neighborhood or community would cause the most concern about higher cost of living?” Respondents selected 1 of the following options: new sidewalks or stop signs, new bicycle lanes or paths, expanded public transportation, new businesses with condos above, improved parks and recreational facilities, or none of these would cause concern.

### Sociodemographic and health characteristics

Respondents self-reported sociodemographic and economic characteristics including sex (male, female), age category (18–34, 35–49, 50–64, ≥65 years), education (high school graduate or less, some college, college graduate or more), race and ethnicity (Hispanic/Latino, non-Hispanic Black, non-Hispanic White, other non-Hispanic [including multiracial]), annual household income (<$50,000, $50,000–$99,999, ≥$100,000), current employment status (working, retired or not working), and housing type (one family house, apartment or other). ConsumerStyles’ database provided geographic information on US Census region (Northeast, Midwest, South, West) and metropolitan statistical area status (nonmetropolitan, metropolitan) (19).

Respondents also self-reported health behaviors (aerobic physical activity and smoking status), anthropometry (height and weight), and a health behavior–related decision about air pollution. To assess physical activity, we used modified versions of the National Health Interview Survey physical activity questions (20). We asked respondents how often in a usual week and, if applicable, the amount of time during leisure time that they participated for at least 10 minutes at a time in 1) vigorous-intensity activities (ie, heavy sweating or large increases in breathing or heart rate) and 2) moderate-intensity activities (ie, medium sweating or moderate increase in breathing or heart rate). To classify adults into levels of physical activity, we calculated minutes of moderate-intensity equivalent activity by counting 1 minute of vigorous-intensity activity as 2 minutes of moderate-intensity activity (1). We then classified respondents into 3 activity levels by using the current adult aerobic guideline (1): 1) active, reporting at least 150 minutes per week of moderate-intensity equivalent physical activity; 2) insufficiently active, reporting some moderate-intensity equivalent physical activity but not enough to meet active definition; and 3) inactive, reporting no moderate-intensity equivalent physical activity that lasted at least 10 minutes. We assessed smoking status by using 2 questions, one about lifetime cigarette use and one about current cigarette use. We combined these and classified respondents into 3 categories: 1) current smoker (respondents who self-reported having smoked at least 100 cigarettes in their lifetime and currently smoked some days or every day); 2) former smoker (respondents who reported having smoked at least 100 cigarettes in their lifetime and currently smoked not at all); and 3) never smoker (respondents who reported having smoked fewer than 100 cigarettes in their lifetime). We used self-reported anthropometry to calculate body mass index (BMI, calculated as weight in kilograms divided by the square of height in meters) and categorized respondents by using standard cut points (21): 1) underweight/normal (<25.0); 2), overweight (25.0–29.9), and 3) obesity (≥30.0). Finally, we asked respondents about decisions related to air pollution exposure by using the question, “When walking, biking, or exercising outdoors, how often do you avoid busy roads to reduce your exposure to air pollution?” (always, usually, sometimes, rarely, never, don’t know).

### Statistical analyses

We calculated descriptive statistics (weighted and unweighted) for all sociodemographic and health characteristics. We calculated prevalence and 95% CIs for the following: 1) agreement that neighborhood development or revitalization has caused concerns about higher cost of living, 2) support for active living improvements even if they lead to a higher cost of living, and 3) specific changes in neighborhood transportation-related and land use–related features. We stratified prevalences by respondent characteristics. We tested assocations between concern, support, and neighborhood features and respondent characteristics by using adjusted Wald χ^2^ tests. Where appropriate, we used pairwise *t* tests with a Bonferroni correction and orthogonal polynomial contrasts to identify significant pairwise differences and trends by participant characteristics. We considered tests significant at *P* < .05, Bonferroni adjusted. All analyses were conducted in 2020 by using SUDAAN release 11 (RTI International) to account for survey weights.

## Results

The largest unweighted percentages of respondents for each demographic group were women, non-Hispanic White, 50 to 64 years old, currently employed, living in a 1-family house, and living in a nonmetropolitan area (Table 1). Slightly more than half were active and never smoked, although more than half were overweight or had obesity. Almost half attempted to reduce air pollution exposure when walking, biking, or exercising outdoors by avoiding busy roads.

**Table 1:** Characteristics of Analytic Sample of US Adults (N = 3,782), SummerStyles Survey, 2018

Characteristics	No. (%)	Weighted % (95% CI)^a^
**Sex**		
Men	1,887 (49.9)	48.6 (46.7–50.4)
Women	1,895 (50.1)	51.4 (49.6–53.3)
**Age, y**		
18–34	690 (18.2)	29.4 (27.5–31.4)
35–49	995 (26.3)	24.1 (22.6–25.6)
50–64	1,242 (32.8)	26.5 (25.0–28.0)
≥65	855 (22.6)	20.1 (18.8–21.4)
**Education level**		
High school graduate or less	1,239 (32.8)	38.7 (36.9–40.6)
Some college	1,101 (29.1)	28.8 (27.2–30.5)
College graduate or more	1,442 (38.1)	32.4 (30.8–34.1)
**Race and ethnicity**		
Black, non-Hispanic	318 (8.4)	10.9 (9.8–12.2)
Hispanic/Latino	353 (9.3)	15.6 (14.0–17.2)
Other, non-Hispanic (including multiracial)	281 (7.4)	8.2 (7.1–9.4)
White, non-Hispanic	2,830 (74.8)	65.3 (63.4–67.2)
**Annual household income, $**		
<50,000	1,171 (31.0)	33.8 (32.0–35.6)
50,000–99,999	1,238 (32.7)	32.6 (30.9–34.4)
≥100,000	1,373 (36.3)	33.6 (32.0–35.3)
**Current employment status**		
Working	2,390 (63.2)	61.5 (59.7–63.3)
Retired or not working	1,392 (36.8)	38.5 (36.7–40.3)
**Housing type**		
One family house	3,146 (83.2)	80.5 (78.9–82.0)
Apartment or other	636 (16.8)	19.5 (18.0–21.1)
**Census region^b^**		
Northeast	709 (18.7)	17.9 (16.6–19.4)
Midwest	841 (22.2)	20.9 (19.5–22.4)
South	1,380 (36.5)	37.5 (35.7–39.3)
West	852 (22.5)	23.6 (22.1–25.3)
**Metropolitan statistical area (MSA) status^c^**		
Nonmetropolitan	3,225 (85.3)	86.0 (84.7–87.2)
Metropolitan	557 (14.7)	14.0 (12.8–15.3)
**Aerobic physical activity level^d^**		
Inactive	562 (14.9)	15.5 (14.2–16.9)
Insufficiently active	1,079 (28.5)	28.5 (26.9–30.2)
Active	2,141 (56.6)	56.0 (54.2–57.8)
**Smoking status^e^**		
Current smoker	426 (11.3)	11.6 (10.5–12.8)
Former smoker	1,143 (30.2)	27.1 (25.6–28.6)
Never smoker	2,213 (58.5)	61.3 (59.6–63.1)
**Body mass index^f^**		
Underweight/normal	1,184 (31.3)	34.0 (32.3–35.9)
Overweight	1,321 (34.9)	32.8 (31.1–34.5)
Obesity	1,277 (33.8)	33.2 (31.5–34.9)
**How often respondent avoids busy roads to reduce exposure to air pollution exposure when walking, biking, or exercising outdoors**		
Always, usually, sometimes	1,885 (49.8)	49.3 (47.1–51.1)
Rarely, never	1,518 (40.2)	40.0 (38.2–41.8)
Don’t know	379 (10.0)	10.7 (9.6–12.0)

a Weighted to the total US population as estimated by the 2018 Current Population Survey by sex, age, annual household income, race and ethnicity, household size, education, census region, and MSA status.

b Regions are defined as the following: Northeast: Connecticut, Maine, Massachusetts, New Hampshire, Rhode Island, New Jersey, New York, Pennsylvania, and Vermont; Midwest: Illinois, Indiana, Iowa, Kansas, Michigan, Minnesota, Missouri, Nebraska, North Dakota, Ohio, South Dakota, and Wisconsin; South: Alabama, Arkansas, Delaware, Florida, Georgia, Kentucky, Louisiana, Mississippi, Maryland, North Carolina, Oklahoma, South Carolina, Virginia, Tennessee, Texas, West Virginia, and District of Columbia; West: Alaska, Arizona, California, Colorado, Hawaii, Idaho, Montana, Nevada, New Mexico, Oregon, Utah, Washington, and Wyoming.

c An MSA was categorized as metropolitian if it was associated with at least 1 urbanized area that has a population of at least 50,000.

d Respondents were classified into 3 activity levels by using the current adult aerobic guideline (1): 1) active, reporting at least 150 min/week of moderate-intensity equivalent physical activity; 2) insufficiently active, reporting some moderate-intensity equivalent physical activity but not enough to meet active definition; and 3) inactive, reporting no moderate-intensity equivalent physical activity that lasted at least 10 min.

e Current smoker: respondents who self-reported having smoked at least 100 cigarettes in their lifetime and currently smoked some days or every day; former smoker: respondents who reported having smoked at least 100 cigarettes in their lifetime, and currently smoked not at all; and never smoker: respondents who reported having smoked fewer than 100 cigarettes in their lifetime.

f Calculated as weight in kilograms divided by the square of height in meters. Underweight/normal: <25.0; overweight: 25.0–29.9; and obesity: ≥30.0.

Almost 1 in 5 respondents reported that development or revitalization had caused concerns about higher cost of living in their neighborhood (19.1%; 95% CI, 17.7%–20.6%) (Table 2). Concern decreased with increasing age and increased with increasing education and physical activity levels. Concern was more prevalent among respondents who were Hispanic/Latino or other non-Hispanic (including multiracial) versus non-Hispanic White; were currently employed versus retired or not working; lived in nonmetropolitan versus metropolitan areas; and lived in the West versus other regions.

**Table 2:** Characteristics of Analytic Sample of US Adults (N = 3,782), by Agreement That Neighborhood Development or Revitalization Has Caused Concerns About Higher Cost of Living, SummerStyles Survey, 2018^a^

Characteristics	Agree, % (95% CI)	Do not agree, % (95% CI)	χ^2^ *P* value
**Total**	19.1 (17.7–20.6)	80.9 (79.4–82.3)	NA
**Sex**			
Men	19.6 (17.6–21.8)	80.4 (78.2–82.4)	.53
Women	18.6 (16.6–20.8)	81.4 (79.2–83.4)	
**Age, y**			
18–34	21.9 (18.6–25.6)^b^	78.1 (74.4–81.4)	<.001
35–49	20.7 (18.0–23.7)	79.3 (76.3–82.0)	
50–64	18.5 (16.2–21.0)	81.5 (79.0–83.8)	
≥65	13.8 (11.5–16.6)	86.2 (83.4–88.5)	
**Education level**			
High school graduate or less	15.7 (13.5–18.3)^b^	84.3 (81.7–86.5)	<.001
Some college	19.9 (17.3–22.8)	80.1 (77.2–82.7)	
College graduate or more	22.4 (20.0–25.0)	77.6 (75.0–80.0)	
**Race and ethnicity**			
Black, non-Hispanic	21.4 (16.8–26.7)^c^^,^ d	78.6 (73.3–83.2)	<.001
Hispanic/Latino	28.9 (23.9–34.4)^d^	71.1 (65.6–76.1)	
Other, non-Hispanic (including multiracial)	25.5 (20.0–31.9)^d^	74.5 (68.1–80.0)	
White, non-Hispanic	15.6 (14.2–17.1)^c^	84.4 (82.9–85.8)	
**Annual household income, $**			
<50,000	19.4 (16.8–22.3)	80.6 (77.7–83.2)	.76
50,000–99,999	19.6 (17.1–22.3)	80.4 (77.7–82.9)	
≥100,000	18.4 (16.1–20.8)	81.6 (79.2–83.9)	
**Current employment status**			
Working	20.6 (18.8–22.6)	79.4 (77.4–81.2)	.01
Retired or not working	16.7 (14.5–19.1)	83.3 (80.9–85.5)	
**Housing type**			
One family house	17.1 (15.6–18.7)	82.9 (81.3–84.4)	<.001
Apartment or other	27.4 (23.6–31.7)	72.6 (68.3–76.4)	
**Region^f^**			
Northeast	20.6 (17.2–24.4)^c^	79.4 (75.6–82.8)	<.001
Midwest	12.8 (10.5–15.7)^d^	87.2 (84.3–89.5)	
South	15.7 (13.7–18.0)^c^	84.3 (82.0–86.3)	
West	28.9 (25.4–32.7)^e^	71.1 (67.3–74.6)	
**Metropolitan statistical area (MSA) status^g^**			
Nonmetropolitan	20.6 (19.0–22.3)	79.4 (77.7–81.0)	<.001
Metropolitan	9.8 (7.3–12.9)	90.2 (87.1–92.7)	
**Aerobic physical activity level^h^**			
Inactive	14.1 (11.1–17.7)^b^	85.9 (82.3–88.9)	.003
Insufficiently active	18.4 (15.8–21.2)	81.6 (78.8–84.2)	
Active	20.9 (18.9–23.0)	79.1 (77.0–81.1)	
**Smoking status^i^**			
Current smoker	18.5 (14.7–23.1)	81.5 (76.9–85.3)	.14
Former smoker	17.0 (14.7–19.6)	83.0 (80.4–85.3)	
Never smoker	20.2 (18.2–22.2)	79.8 (77.8–81.8)	
**Body mass index^j^**			
Underweight/normal	19.4 (16.9–22.2)	80.6 (77.8–83.1)	.89
Overweight	18.6 (16.3–21.2)	81.4 (78.8–83.7)	
Obesity	19.3 (16.8–22.0)	80.7 (78.0–83.2)	
**How often respondent avoids busy roads to reduce exposure to air pollution when walking, biking, or exercising outdoors**			
Always, usually, sometimes	22.0 (19.9–24.2)^c^	78.0 (75.8–80.1)	<.001
Rarely, never	17.0 (14.9–19.5)^d^	83.0 (80.5–85.1)	
Don’t know	13.5 (9.8–18.3)^e^	86.5 (81.7–90.2)	
**Support changes to make it easier to walk or bike even if they lead to a higher cost of living**			
Supporters	24.6 (22.4–26.9)^c^	75.4 (73.1–77.6)	<.001
Nonsupporters	19.7 (16.3–23.5)^c^	80.3 (76.5–83.7)	
Neither	10.5 (8.6–12.6)^d^	89.5 (87.4–91.4)	

Abbreviation: NA, not applicable.

a Weighted to the total US population as estimated by the annual Current Population Survey by sex, age, annual household income, race and ethnicity, household size, education, census region, and MSA status.

b Signifiant linear trend, using orthogonal polynomial contrasts for trends test.

c, d, e Values within a column and in the same category that do not share a common superscripted letter are significantly different (Bonferroni corrected *P* < .05), whereas values that do share a common superscripted letter are not significantly different, using pairwise *t* tests.

f Regions are defined as the following: Northeast: Connecticut, Maine, Massachusetts, New Hampshire, Rhode Island, New Jersey, New York, Pennsylvania, and Vermont; Midwest: Illinois, Indiana, Iowa, Kansas, Michigan, Minnesota, Missouri, Nebraska, North Dakota, Ohio, South Dakota, and Wisconsin; South: Alabama, Arkansas, Delaware, Florida, Georgia, Kentucky, Louisiana, Mississippi, Maryland, North Carolina, Oklahoma, South Carolina, Virginia, Tennessee, Texas, West Virginia, and District of Columbia; West: Alaska, Arizona, California, Colorado, Hawaii, Idaho, Montana, Nevada, New Mexico, Oregon, Utah, Washington, and Wyoming.

g An MSA was categorized as metropolitian if it was associated with at least 1 urbanized area that has a population of at least 50,000.

h Respondents were classified into 3 activity levels by using the current adult aerobic guideline (1): 1) active, reporting at least 150 min/week of moderate-intensity equivalent physical activity; 2) insufficiently active, reporting some moderate-intensity equivalent physical activity but not enough to meet active definition; and 3) inactive, reporting no moderate-intensity equivalent physical activity that lasted at least 10 min.

i Current smoker: respondents who self-reported having smoked at least 100 cigarettes in their lifetime and currently smoked some days or every day; former smoker: respondents who reported having smoked at least 100 cigarettes in their lifetime, and currently smoked not at all; and never smoker: respondents who reported having smoked fewer than 100 cigarettes in their lifetime.

j Calculated as weight in kilograms divided by the square of height in meters. Underweight/normal: <25.0; overweight: 25.0–29.9; and obesity: ≥30.0.

Overall, approximately half of respondents (50.7%; 95% CI, 48.9%–52.6%) supported changes to make it easier to walk or bike even if they lead to a higher cost of living (Table 3). Respondents who reported concerns about higher cost of living in their neighborhood were more likely to express support (65.3%) than respondents who did not report concerns (47.3%). Similar to the prevalence of concerns about neighborhood development, the prevalence of support decreased with increasing age and increased with increasing education and physical activity level. The prevalence of support also increased with increasing income and decreased with increasing BMI. We found no association between race or ethnicity and support for changes to make it easier to walk or bike even if they lead to a higher cost of living. Unlike concern, support did not vary by employment status, housing type, or region.

**Table 3:** Characteristics of Analytic Sample of US Adults (N = 3,782), by Support for Changes to Make It Easier to Walk or Bike Even if They Lead to a Higher Cost of Living, SummerStyles Survey, 2018^a^

Characteristics	Supporters, % (95% CI)	Nonsupporters, % (95% CI)	Neither, % (95% CI)	χ^2^ *P* value
**Total**	50.7 (48.9–52.6)	16.1 (14.8–17.4)	33.2 (31.5–35.0)	NA
**Sex**				
Men	50.6 (48.0–53.2)	17.1 (15.2–19.1)	32.3 (29.9–34.8)	.32
Women	50.8 (48.2–53.4)	15.1 (13.4–17.1)	34.0 (31.6–36.6)	
**Age, y**				
18–34	51.0 (46.7–55.2)^b^	15.0 (12.2–18.2)	34.1 (30.1–38.2)	.01
35–49	54.2 (50.8–57.6)	16.4 (14.1–19.1)	29.4 (26.3–32.6)	
50–64	51.2 (48.2–54.2)	16.7 (14.6–19.0)	32.1 (29.4–35.0)	
≥65	45.5 (42.0–49.0)	16.5 (14.0–19.3)	38.0 (34.6–41.6)	
**Education level**				
High school graduate or less	40.6 (37.5–43.9)^b^	16.8 (14.5–19.3)^b^	42.6 (39.4–45.8)^b^	<.001
Some college	51.5 (48.2–54.9)	18.2 (15.8–20.9)	30.3 (27.3–33.4)	
College graduate or more	62.1 (59.2–64.8)	13.3 (11.6–15.3)	24.6 (22.2–27.2)	
**Race and ethnicity**				
Black, non-Hispanic	55.4 (49.3–61.3)	13.5 (9.7–18.4)	31.2 (25.8–37.1)	.03
Hispanic/Latino	56.2 (50.4–61.8)	12.9 (9.5–17.2)	30.9 (25.8–36.5)	
Other, non-Hispanic (including multiracial)	47.3 (40.3–54.4)	13.5 (9.4–19.1)	39.2 (32.3–46.6)	
White, non-Hispanic	49.1 (47.0–51.1)	17.6 (16.1–19.2)	33.4 (31.4–35.3)	
**Annual household income, $**				
<50,000	42.2 (38.9–45.5)^b^	17.6 (15.2–20.2)	40.3 (37.0–43.6)^b^	<.001
50,000–99,999	52.2 (49.0–55.4)	15.8 (13.6–18.2)	32.0 (29.1–35.1)	
≥100,000	57.9 (54.8–60.8)	14.8 (12.9–17.1)	27.3 (24.6–30.2)	
**Current employment status**				
Working	55.3 (53.0–57.6)^c^	15.8 (14.3–17.5)	28.8 (26.8–31.0)^c^	<.001
Retired or not working	43.3 (40.4–46.4)^d^	16.4 (14.3–18.8)	40.2 (37.2–43.3)^d^	
**Housing type**				
One family house	52.3 (50.3–54.3)^c^	15.7 (14.3–17.2)	32.0 (30.2–34.0)^c^	.005
Apartment or other	44.3 (39.9–48.7)^d^	17.6 (14.4–21.4)	38.1 (33.9–42.5)^d^	
**Region^f^**				
Northeast	48.9 (44.7–53.1)	16.0 (13.3–19.2)	35.1 (31.0–39.3)	.14
Midwest	48.6 (44.7–52.5)	14.8 (12.4–17.6)	36.6 (32.9–40.5)	
South	53.3 (50.3–56.3)	15.2 (13.2–17.5)	31.5 (28.7–34.4)	
West	49.8 (45.9–53.7)	18.6 (15.7–21.9)	31.6 (28.1–35.3)	
**Metropolitan statistical area (MSA)^g^**				
Nonmetropolitan	51.9 (49.9–53.9)^c^	15.3 (14.0–16.8)^c^	32.7 (30.9–34.7)	.002
Metropolitan	43.2 (38.7–47.8)^d^	20.7 (17.1–24.7)^d^	36.1 (31.8–40.7)	
**Aerobic physical activity level^h^**				
Inactive	35.9 (31.5–40.6)^b^	18.4 (15.1–22.3)	45.6 (41.0–50.4)^b^	<.001
Insufficiently active	45.8 (42.4–49.2)	17.6 (15.2–20.2)	36.6 (33.4–40.0)	
Active	57.3 (54.9–59.7)	14.7 (13.0–16.5)	28.0 (25.9–30.3)	
**Smoking status^i^**				
Current smoker	44.5 (39.2–49.9)^c^	19.7 (15.8–24.3)	35.8 (30.9–41.1)	.02
Former smoker	49.5 (46.3–52.7)^c^^,^ d	17.9 (15.7–20.5)	32.6 (29.7–35.6)	
Never smoker	52.4 (50.0–54.9)^d^	14.6 (13.0–16.4)	33.0 (30.7–35.4)	
**Body mass index^j^**				
Underweight/normal	51.6 (48.3–54.9)^b^	14.7 (12.5–17.1)	33.7 (30.6–37.0)	.007
Overweight	53.6 (50.5–56.7)	16.9 (14.8–19.3)	29.4 (26.7–32.3)	
Obesity	46.9 (43.8–50.0)	16.7 (14.5–19.1)	36.4 (33.4–39.5)	
**How often respondent avoids busy roads to reduce exposure to air pollution when walking, biking, or exercising outdoors**				
Always, usually, sometimes	58.6 (56. 0–61.1)^c^	13.8 (12.1–15.6)^c^	27.6 (25.3–30.1)^c^	<.001
Rarely, never	45.9 (43.0–48.8)^d^	19.1 (17.0–21.5)^d^	35.0 (32.2–37.9)^d^	
Don’t know	32.7 (27.5–38.3)^e^	15.2 (11.7–19.6)^c^^,^ d	52.1 (46.4–57.8)^e^	
**Agree that neighborhood is experiencing development or revitalization that has caused concerns about higher cost of living**				
Agree	65.3 (61.1–69.3)^c^	16.6 (13.7–19.9)	18.2 (15.1–21.8)^c^	<.001
Do not agree	47.3 (45.3–49.3)^d^	16.0 (14.6–17.5)	36.8 (34.8–38.8)^d^	

Abbreviation: NA, not applicable.

a Weighted to the total US population as estimated by the annual Current Population Survey by sex, age, annual household income, race and ethnicity, household size, education, census region, and MSA status.

b Signifiant linear trend, using orthogonal polynomial contrasts for trends test.

c, d, e Values within a column and in the same category that do not share a common superscripted letter are significantly different (Bonferroni corrected *P* < .05), whereas values that do share a common superscripted letter are not significantly different, using pairwise *t* tests.

f Regions are defined as the following: Northeast: Connecticut, Maine, Massachusetts, New Hampshire, Rhode Island, New Jersey, New York, Pennsylvania, and Vermont; Midwest: Illinois, Indiana, Iowa, Kansas, Michigan, Minnesota, Missouri, Nebraska, North Dakota, Ohio, South Dakota, and Wisconsin; South: Alabama, Arkansas, Delaware, Florida, Georgia, Kentucky, Louisiana, Mississippi, Maryland, North Carolina, Oklahoma, South Carolina, Virginia, Tennessee, Texas, West Virginia, and District of Columbia; West: Alaska, Arizona, California, Colorado, Hawaii, Idaho, Montana, Nevada, New Mexico, Oregon, Utah, Washington, and Wyoming.

g An MSA was categorized as metropolitian if it was associated with at least 1 urbanized area that has a population of at least 50,000.

h Respondents were classified into 3 activity levels by using the current adult aerobic guideline (1): 1) active, reporting at least 150 min/week of moderate-intensity equivalent physical activity; 2) insufficiently active, reporting some moderate-intensity equivalent physical activity but not enough to meet active definition; and 3) inactive, reporting no moderate-intensity equivalent physical activity that lasted at least 10 min.

i Current smoker: respondents who self-reported having smoked at least 100 cigarettes in their lifetime and currently smoked some days or every day; former smoker: respondents who reported having smoked at least 100 cigarettes in their lifetime, and currently smoked not at all; and never smoker: respondents who reported having smoked fewer than 100 cigarettes in their lifetime.

j Calculated as weight in kilograms divided by the square of height in meters. Underweight/normal: <25.0; overweight: 25.0–29.9; and obesity: ≥30.0.

Of the specific changes in neighborhood features that could cause concern about higher cost of living, new businesses with condos above was the greatest land use–related concern (21.7%; 95% CI, 20.2%–23.3%), followed by improved parks and recreation facilities (8.2%; 95% CI, 7.2%–9.3%) (Figure). Expanded public transportation was the greatest transportation-related concern (8.0%; 95% CI, 7.1%–9.0%). Respondents who reported concern about higher cost of living from neighborhood development overall also had higher prevalence of concern than respondents who did not report concern across all infrastructure types, except new sidewalks or stop signs and improved parks and recreation facilities. Supporters (versus neither) of active living changes reported greatest concern about new businesses and improved parks, while nonsupporters (versus neither) reported greatest concern for new bicycle lanes or paths (Figure). Physical activity and BMI were the only respondent characteristics that were not associated with concerns about specific changes in neighborhood features.

**Figure:**
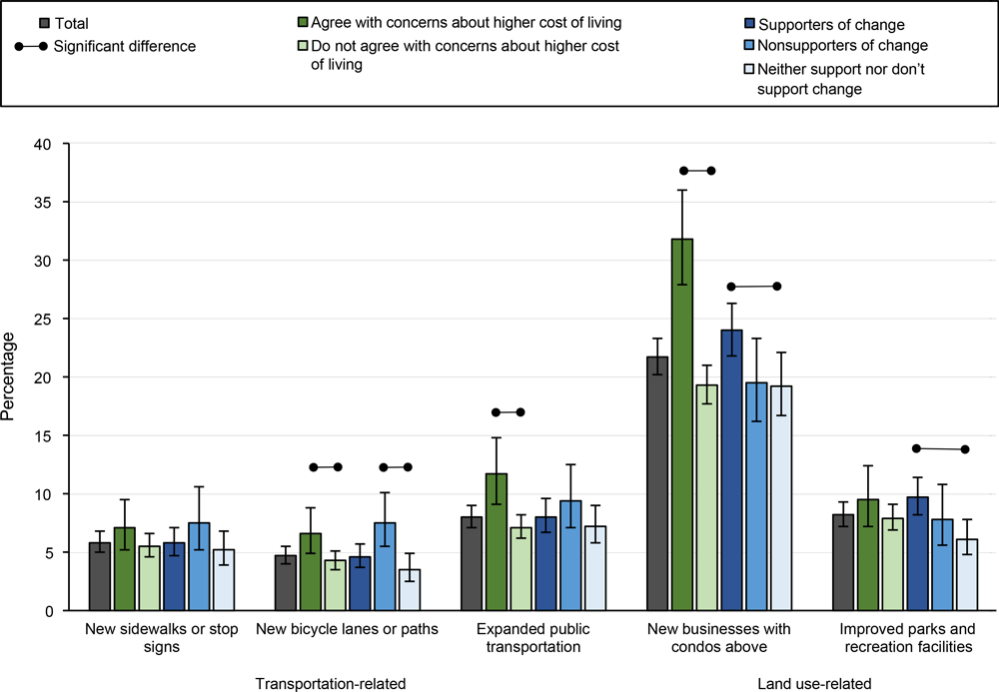
Prevalence of residents reporting specific changes in neighborhood features as causing concern, stratified by agreement with concerns about higher cost of living caused by changes and by support for changes to their neighborhoods even if the changes lead to higher cost of living, SummerStyles survey, 2018 (N = 3,782).

**Table:** 

Category	Transportation-related, % (95% CI)			Land use–related, % (95% CI)	
	New sidewalks or stop signs	New bicycle lanes or paths	Expanded public transportation	New businesses with condos above	Improved parks and recreation facilities
Total	5.8 (5.0–6.8)	4.7 (4.0–5.5)	8.0 (7.1–9.0)	21.7 (20.2–23.3)	8.2 (7.2–9.3)
Agree with concerns for higher cost of living	7.1 (5.2–9.5)	6.6 (4.9–8.8)	11.7 (9.1–14.8)	31.8 (27.9–36.0)	9.5 (7.2–12.4)
Do not agree with concerns for higher cost of living	5.5 (4.6–6.6)	4.3 (3.5–5.1)	7.1 (6.2–8.2)	19.3 (17.7–21.0)	7.9 (6.9–9.1)
Supporters of change	5.8 (4.7–7.1)	4.6 (3.7–5.7)	8.0 (6.7–9.6)	24.0 (21.8–26.3)	9.7 (8.2–11.4)
Nonsupporters of change	7.5 (5.2–10.6)	7.5 (5.5–10.1)	9.4 (7.1–12.5)	19.5 (16.2–23.3)	7.8 (5.6–10.8)
Neither support nor don’t support change	5.2 (3.9–6.8)	3.5 (2.5–4.9)	7.2 (5.8–9.0)	19.2 (16.7–22.1)	6.1 (4.8–7.8)

For new bicycle lanes or paths, there is a significant difference between agree with concerns about higher cost of living and do not agree with concerns about higher cost of living and between nonsupporters of change and neither support nor don’t support change. For expanded public transportation, there is a significant difference between agree with concerns about higher cost of living and do not agree with concerns about higher cost of living. For new businesses with condos above, there are significant differences between agree with concerns about higher cost of living and do not agree with concerns about higher cost of living and between supporters of change and nonsupporters of change. For improved parks and recreation facilities, there is a significant difference between nonsupporters of change and neither support nor don’t support change.

## Discussion

This study leveraged data from a nationwide consumer panel survey to better understand perceptions of community development and revitalization strategies and whether residents were concerned about cost of living increases resulting from built environment changes to improve health. Results showed substantial levels of support for health-promoting neighborhood improvements in potential contradiction with concerns about increased costs among some of the same demographic groups. Respondents who reported concerns about development raising costs were more likely to support changes to make it easier to walk or bike even if they led to increased costs (65.3%) than respondents who did not report such concerns (47.3%). However, given that a small percentage of the population reported concerns to begin with, these inconsistencies are not highly prevalent in the overall population. Certain types of built environment changes, such as new businesses with condos above, were associated with more concern than other types of built environment changes, such as new sidewalks or stop signs. Understanding that residents may be both supportive and concerned, as well as understanding the sources of concern, may be useful for decision makers as they seek to build community support for built environmental changes to improve active living.

Cost of living is a concern among US residents (22). However, these results offer only limited support for our hypothesis that populations disproportionately affected by increases in cost would express more concern about and be less likely to support built environment changes to increase physical activity. In particular, the finding that support for changes to make it easier to walk or bike despite increasing costs was more prevalent among respondents reporting concerns compared with those not reporting concerns about development raising costs ran counter to our hypothsis. Survey results depicted greater concern among respondents living in the West, potentially because that area is developing and urbanizing more rapidly than other parts of the US (23). Consistent with previous SummerStyles analyses, our analysis found that respondents with higher education levels expressed increased concern about cost of living changes resulting from neighborhood development and revitalization (24).

More than half of study respondents supported built environment or infrastructural changes to promote active living even if the changes could lead to a higher cost of living, despite nearly 1 in 5 reporting concerns about such changes leading to increases in cost of living. This is consistent with another national survey examining support for policies that promote physical activity in neighborhood environments even when these policies are associated with tax increases (25). However, the present survey failed to confirm our hypothesis that low-income or racial or ethnic minority populations would not support environmental changes to improve active living.

Although this support from low-income or racial or ethnic minority populations may seem counterintuitive, other studies have discussed the complexity and nuances of perceptions of neighborhood revitalization and development, with residents expressing support while acknowledging a “not for us” sentiment (26). Additional investigation of the complexities of resident concerns and support for changes in the active living environment could guide implementation of active living improvements and help communities avoid unintended consequences. Involving community members meaningfully in neighborhood revitalization processes could also enhance understanding of these complexities. Further research could clarify whether inclusive policies could help ensure that economic development benefits existing residents who have often been historically disinvested and excluded (27).

When examining specific transportation-related and land use–related neighborhood changes that could cause concern about a higher cost of living, respondents were more likely to be concerned about changes representing larger investments, such as new businesses with condos above, improved parks and recreation facilities, and expanded urban transit. These sizable urban developments also have considerable economic implications and have been documented to draw businesses and new residents that can change neighborhood character, potentially resulting in gentrification and displacement of previous businesses and residents (28). In contrast, environmental changes such as new sidewalks and stop signs or new bicycle lanes and paths may be perceived as smaller investments, potentially driven by safety versus economic concerns and thus less likely to indicate changes in an area’s economic opportunities.

### Limitations and strengths

This study has some important limitations. One is potential sample selection bias associated with data from a volunteer-based panel survey. Although the sample had nationwide representation, people who agreed to participate may be different than those who did not, which might potentially bias results (29). Sample selection bias could also result from address-based sampling that does not reach homeless or institutionalized populations, which could potentially skew results toward higher-resource populations (29). In addition, data were self-reported and may be affected by recall bias (29). Survey responses may also be affected by social desirability bias, or the inclination to frame behaviors or attitudes in a positive manner, especially regarding physical activity behaviors and support for active living infrastructural changes. Survey questions were also not cognitively tested or psychometrically assessed before administration, so insight is lacking about respondents’ interpretation of survey question phrasing, such as “new businesses with condos above.” In particular, the survey question about concerns did not specify perceived concerns for the individual but concerns overall, possibly at the neighborhood level, leading to possibility that the individual is supportive despite neighborhood concerns. In addition, respondents may have disagreed with the question about concern if they did not perceive their neighborhood as experiencing development. However, an interpretation focused on cost of living seems more likely, because the question on concern was immediately preceded on the survey by the question on support for changes to make it easier to walk or bike despite higher cost of living. The SummerStyles survey also did not collect data about active or vehicular commutes. This could affect perceptions, especially among those with active commutes who may be more supportive of active transportation infrastructure than others. Lastly, we were unable to differentiate between new residents and long-term residents who may have been aware of previous or planned built environment changes to improve active living, because the survey did not ask the length of time that respondents have lived in their neighborhoods.

This study also has several strengths. Data about perceptions of neighborhood infrastructure to support physical activity with a nationwide sample are rare and a substantial advantage of the survey. Furthermore, no previous study has examined the association between demographic characteristics and perceptions of neighborhood infrastructure to support physical activity. Survey questions parallel those from other widely used built environment assessment tools, such as the Neighborhood Environmental Walkability Scale (30), which facilitates comparison of results across studies. Lastly, the sample size was large, allowing us to examine differences across many different demographic characteristics.

### Conclusion

While support for built environment changes to promote active living differed between demographic groups, this study found support for active living infrastructural changes, despite concerns over increased cost of living. It is important to understand community perceptions about the built environment or infrastructural changes to facilitate active living, because community buy-in and meaningful participation are important for implementation (31). Communities that comprise people with low incomes and other traditionally marginalized demographic groups are historically and currently oppressed by government entities, resulting in a lack of trust in engagement opportunities during the development process (13). As a result, community input may not accurately represent the views of these populations. Future research should aim to articulate best practices for equitable community engagement during the development and implementation processes. Solutions such as participatory planning hold potential for community engagement in decisions about active living infrastructure (32). Policy recommendations abound for mitigating displacement risk, though few have been evaluated (33). Public health and planning professionals can partner with communities to select the most appropriate measures for each context and evaluate each carefully for equity and effectiveness. Studies that further explore complexity in resident perceptions of neighborhood improvements to support active living would help communities respond to concerns when planning changes to promote health. This study has implications for engaging residents in decisions by addressing potential barriers to support for transportation and land use changes focused on increasing active living and physical activity.
